# Correction

**DOI:** 10.1093/biomethods/bpag030

**Published:** 2026-06-16

This is a correction to: Thang Truong Le, Vinh-Thuyen Nguyen-Truong, Quang Van Nhat Duong, Nghia Trong Le Phan, Phuc Nguyen Thien Dao, Mqondisi Fortune Mavuso, Huy Ngoc Anh Nguyen, Tien Thuy Mai, Kiep Thi Quang, Deep learning-based classification of colorectal cancer in histopathology images for category detection, *Biology Methods and Protocols*, Volume 10, Issue 1, 2025, bpaf077, https://doi.org/10.1093/biomethods/bpaf077

In the originally published version of this manuscript, the institutional affiliation listed for the second co-author contained errors. 

This has now been corrected to read: “John von Neumann Institute, VNUHCM, Vo Truong Toan Street, Ho Chi Minh, 700000, Vietnam” instead of: “John von Neumann Institute, Vietnam National University HCM, Vinh Truong, Binh Duong, 75000, Vietnam”.  

