# Supplementary material for: Deep learning-based classification of colorectal cancer in histopathology images for category detection
Source: Biol Methods Protoc. 2025 Oct 22;10(1):bpaf077. doi: 10.1093/biomethods/bpaf077 (PMC12622963; doi:10.1093/biomethods/bpaf077)
Supplement: bpaf077_Supplementary_Data [file bpaf077_supplementary_data.zip › SUPPLEMENTARY.docx]

**SUPPLEMENTARY**

**S. FIGURE 1.** The representative pathological images for six differentiation categories from EBHI-Seg dataset.

**S. FIGURE 2.** ResNet-18, ResNet-34, and ResNet-50 architecture.

**S. FIGURE 3.** ResNet-18 classification result.

**S. FIGURE 4.** ResNet-50 classification result.

**S. FIGURE 5.** Swin v2-T W8 Evaluation.

**S. FIGURE 6.** ROC AUC OvO of the 2 Swin model.

**S. FIGURE 7.** ResNet model GradCAM explanation on EBHI-Seg dataset.

**S. FIGURE 8.** Swin Transformer model GradCAM explanation on EBHI-Seg dataset.

**S. TABLE 1.** Comparison of models in size (Params) and FLOPs.

| Model | Params (Million) | FLOPs (GFLOPs) |
| --- | --- | --- |
| ResNet 18 | 11.18 | 1.82 |
| ResNet 34 | 21.29 | 3.68 |
| ResNet 50 | 23.52 | 4.13 |
| SwinV2-t-W8 | 27.58 | 4.37 |
| SwinV2-s-W8 | 48.96 | 8.48 |
