# Supplementary figures and images for: Deep learning-based classification of colorectal cancer in histopathology images for category detection
Source: Biol Methods Protoc. 2025 Oct 22;10(1):bpaf077. doi: 10.1093/biomethods/bpaf077 (PMC12622963; doi:10.1093/biomethods/bpaf077)

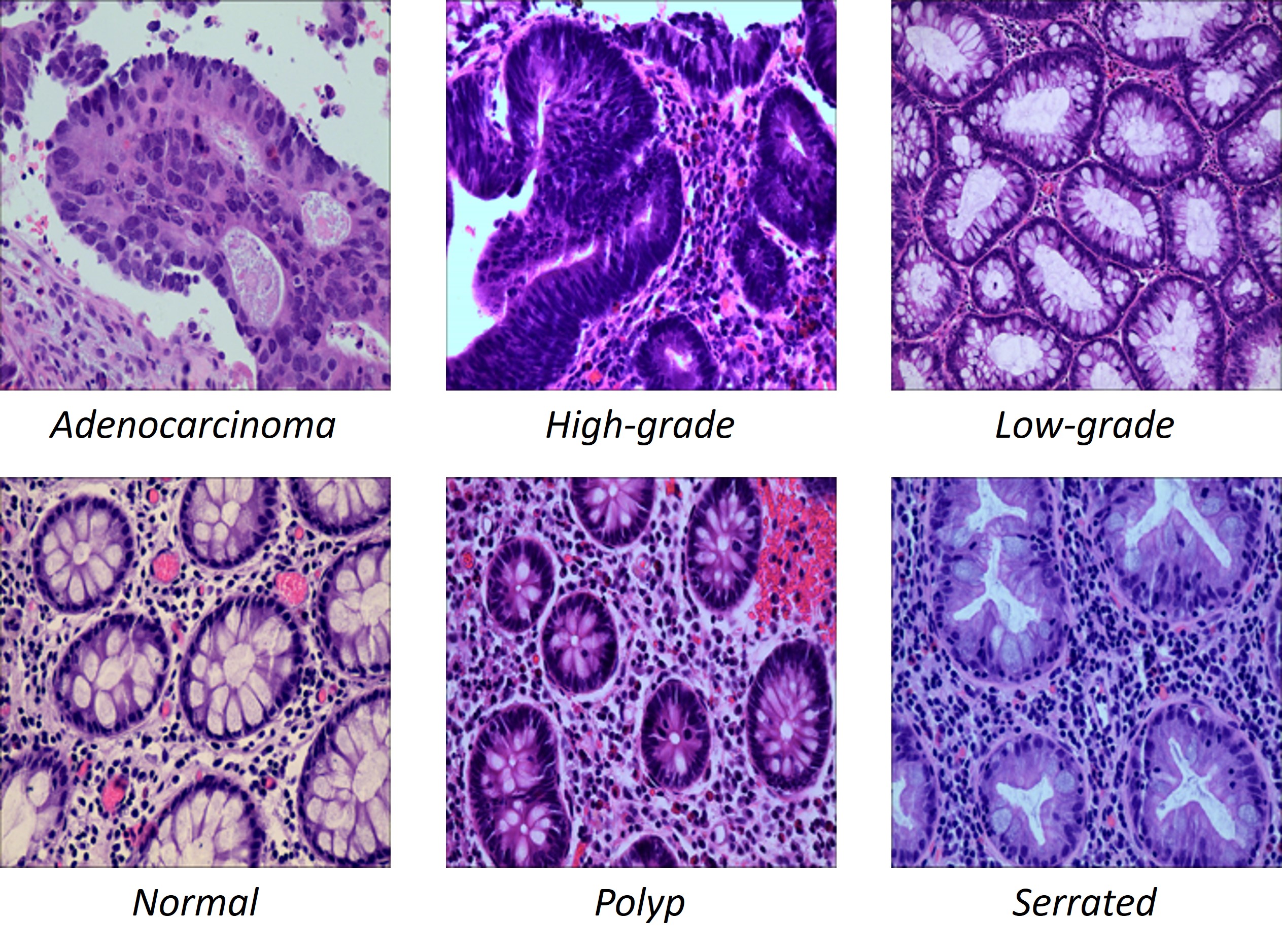

Supplement: bpaf077_Supplementary_Data [file bpaf077_supplementary_data.zip › SFig1.jpg]

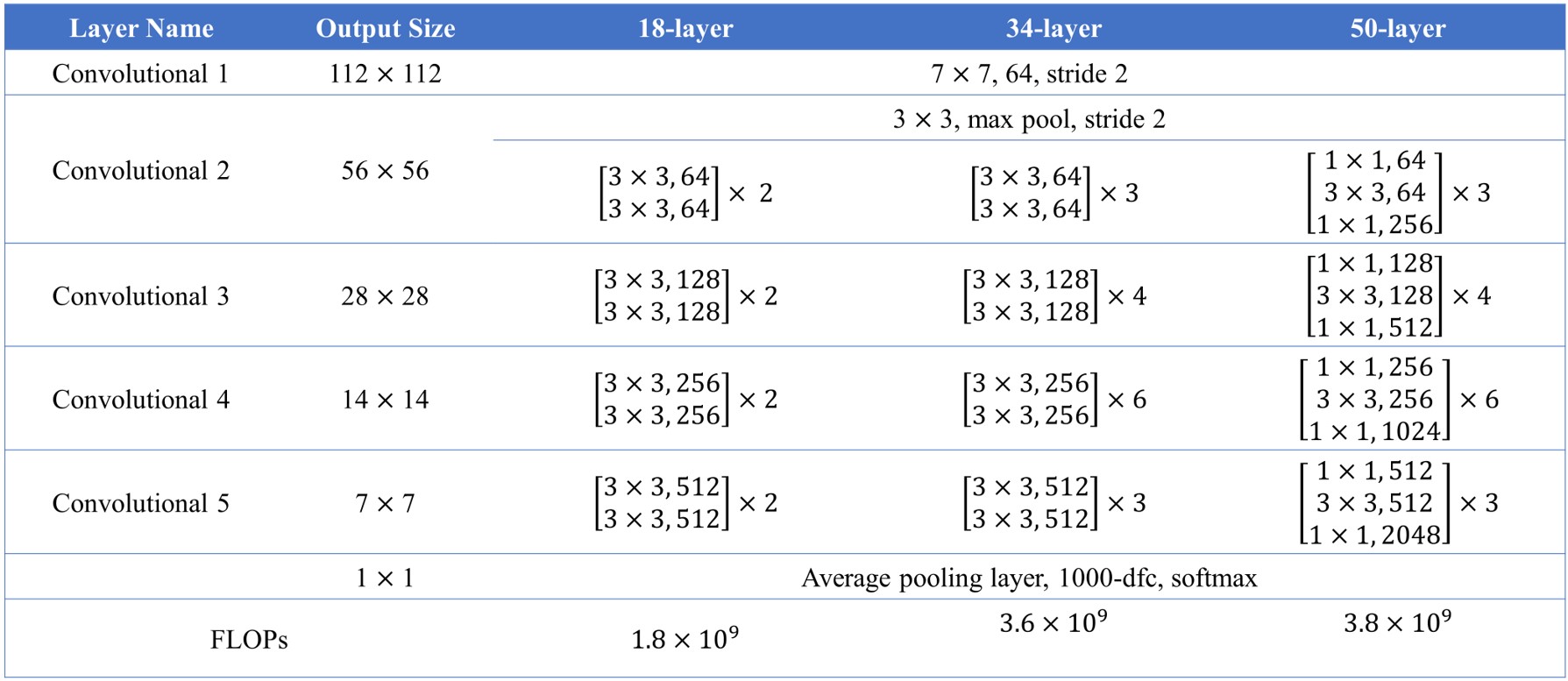

Supplement: bpaf077_Supplementary_Data [file bpaf077_supplementary_data.zip › SFig2.jpg]

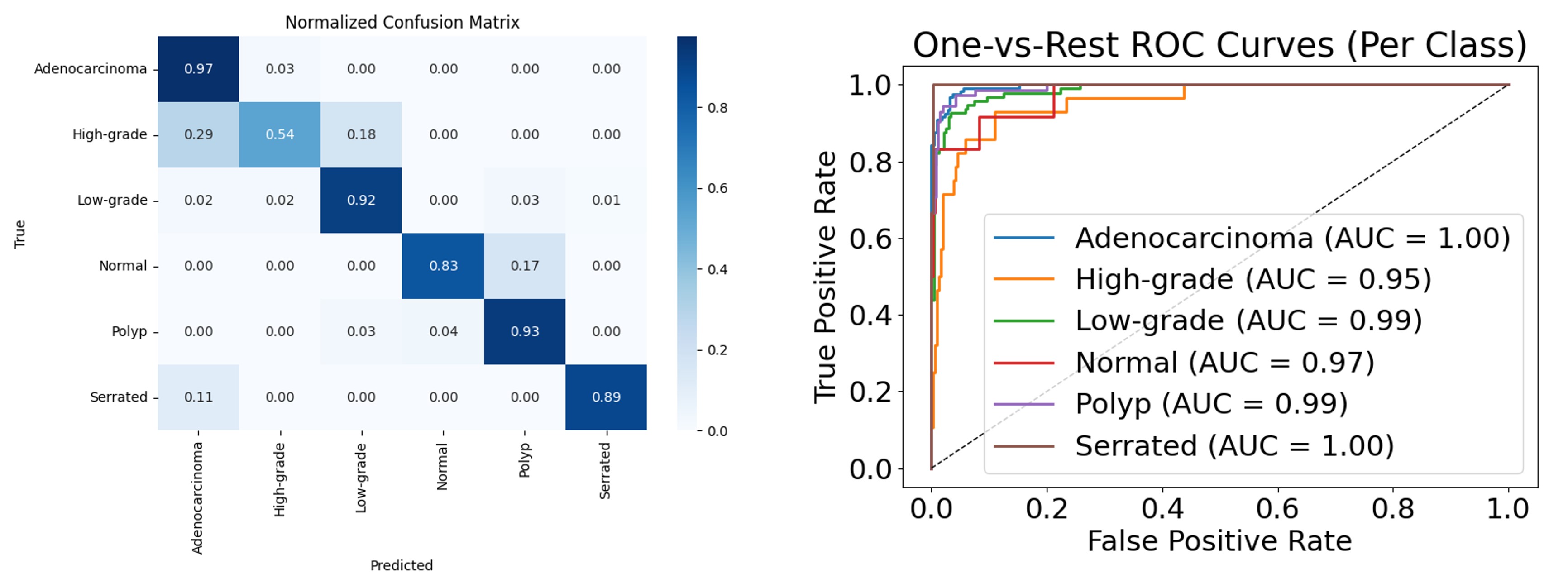

Supplement: bpaf077_Supplementary_Data [file bpaf077_supplementary_data.zip › SFig3.jpg]

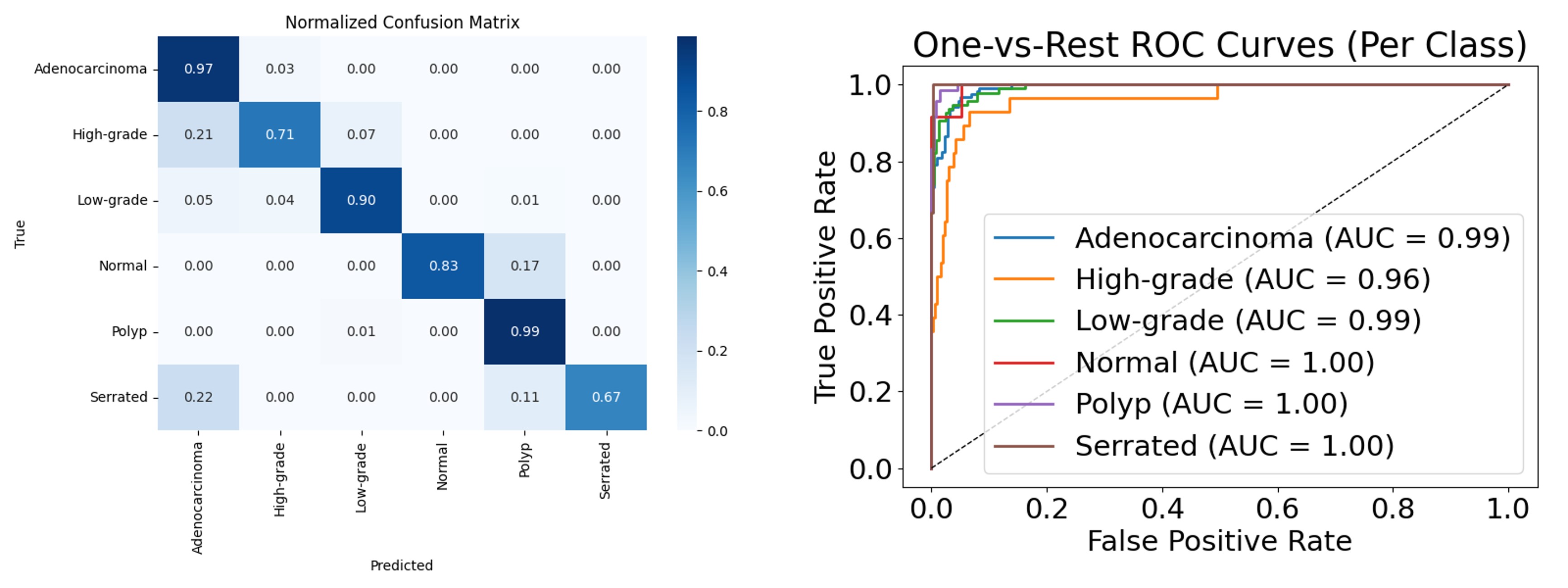

Supplement: bpaf077_Supplementary_Data [file bpaf077_supplementary_data.zip › SFig4.jpg]

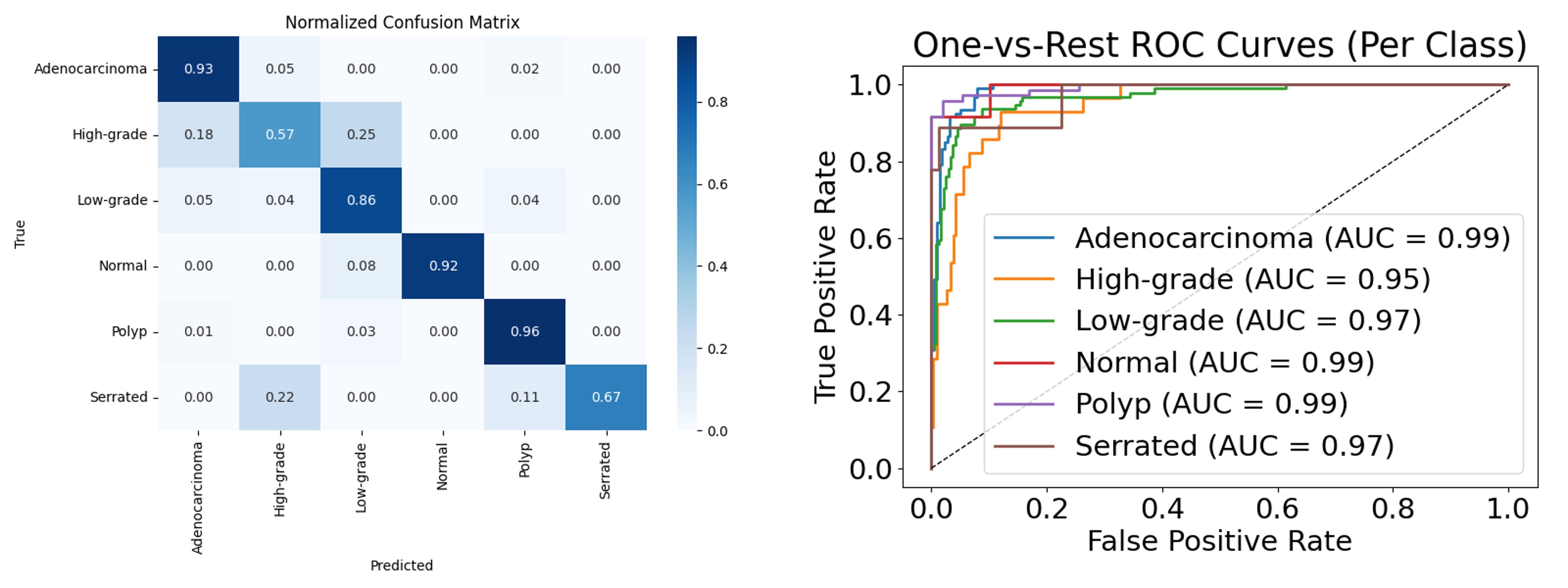

Supplement: bpaf077_Supplementary_Data [file bpaf077_supplementary_data.zip › SFig5.jpg]

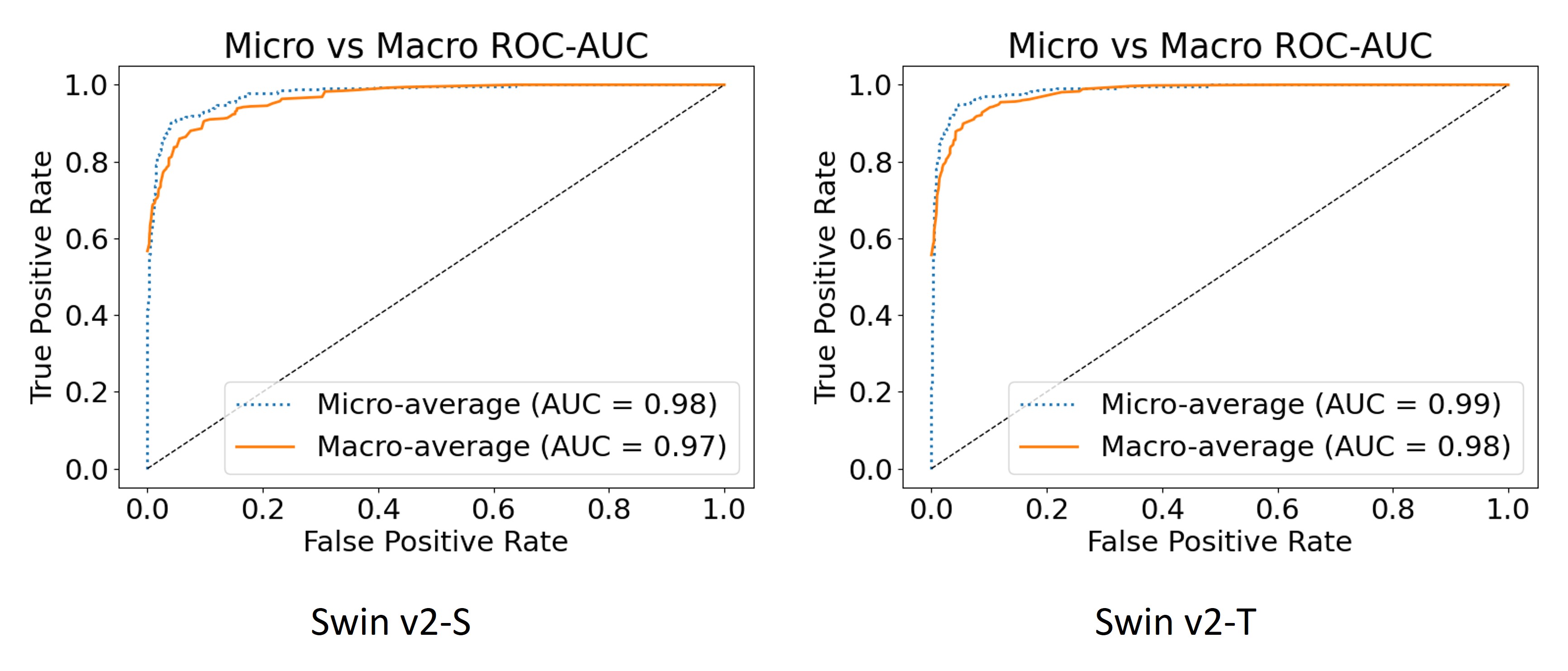

Supplement: bpaf077_Supplementary_Data [file bpaf077_supplementary_data.zip › SFig6.jpg]

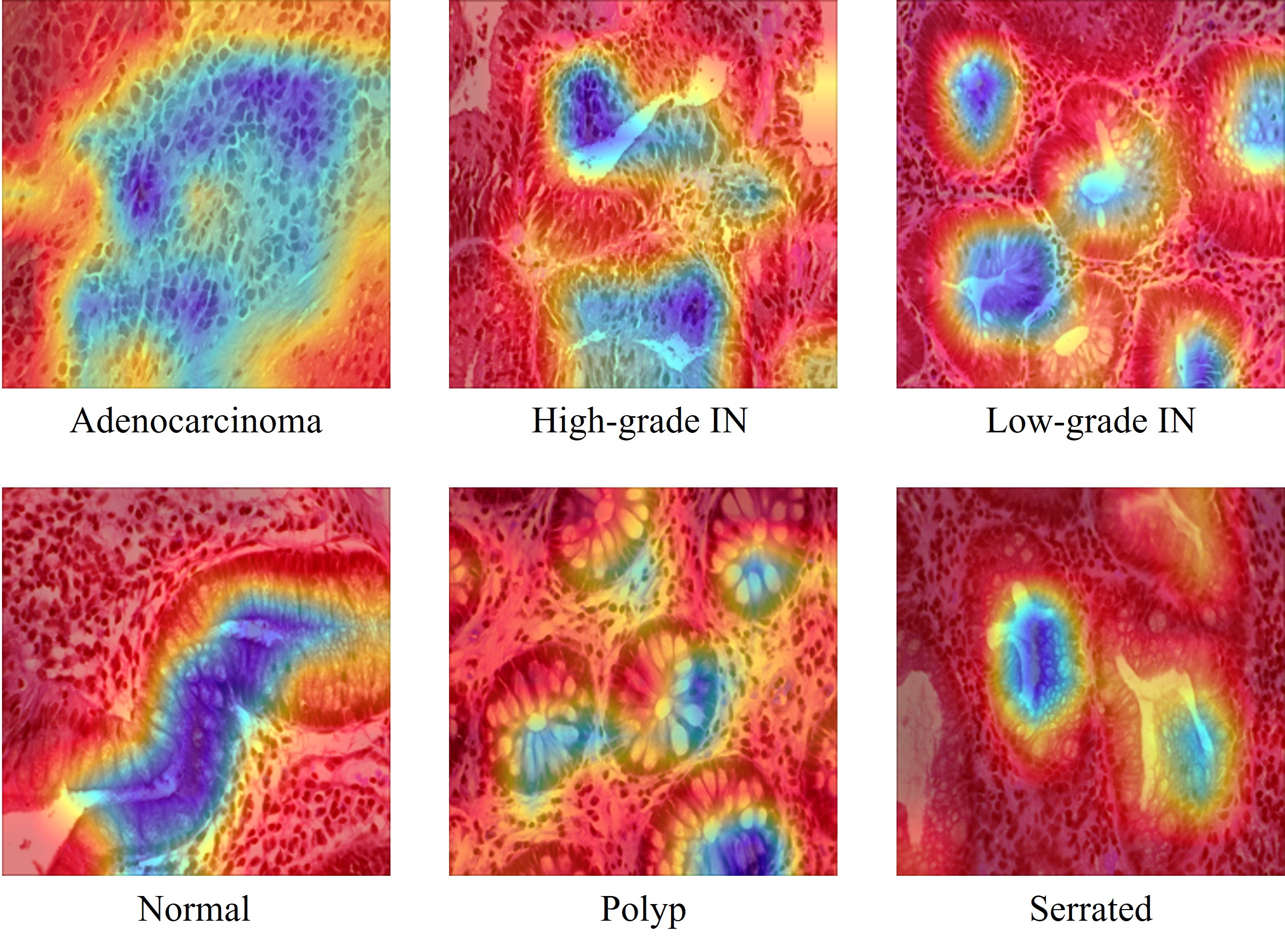

Supplement: bpaf077_Supplementary_Data [file bpaf077_supplementary_data.zip › SFig7.jpg]

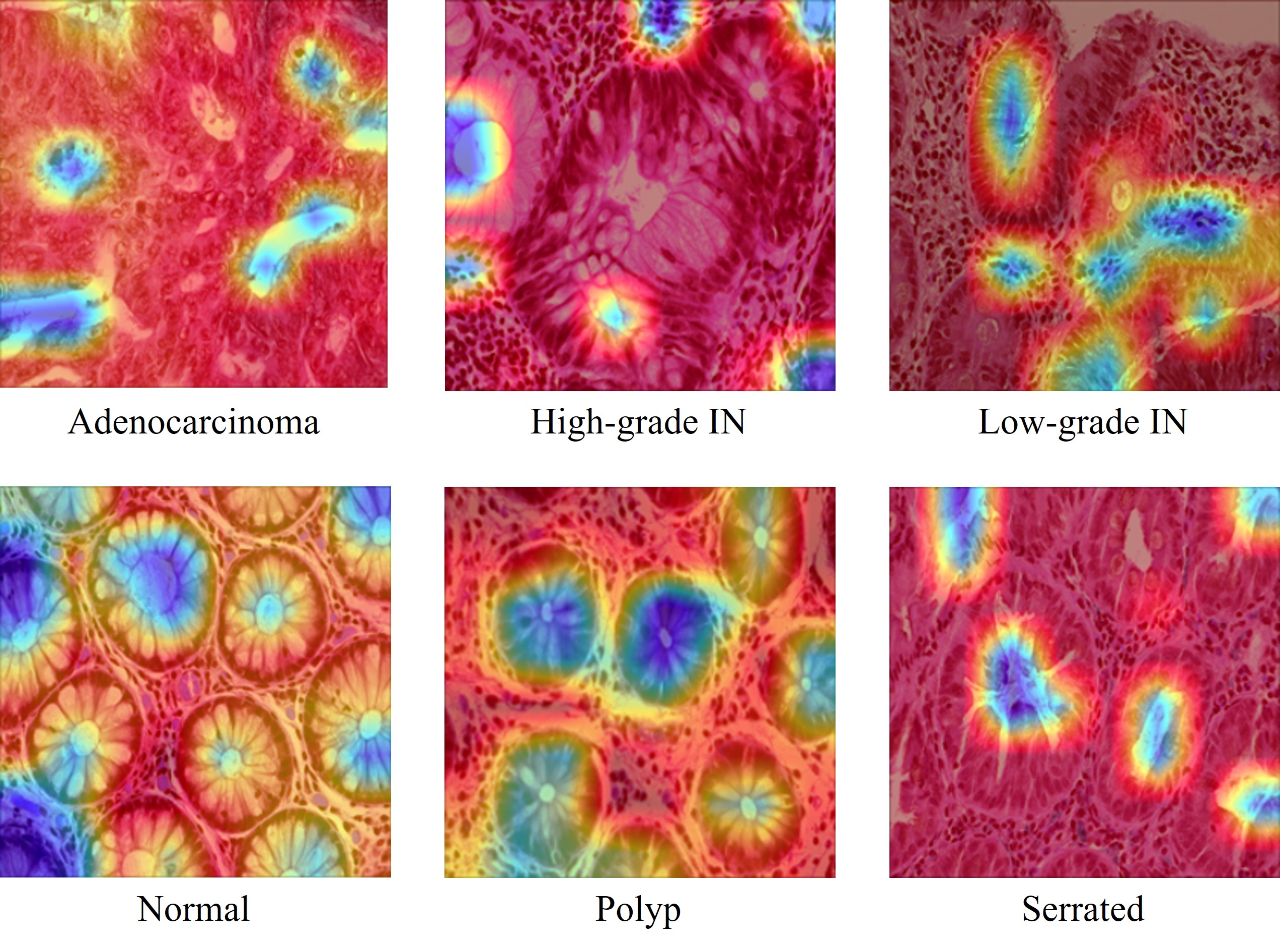

Supplement: bpaf077_Supplementary_Data [file bpaf077_supplementary_data.zip › SFig8.jpg]
